# Bile salt hydrolase activity as a rational target for MASLD therapy

**DOI:** 10.1080/19490976.2025.2608437

**Published:** 2026-01-02

**Authors:** Elizabeth V. Jones, Yongtao Wang, Wenchao Wei, James C. Reed, Snehal N. Chaudhari, Darrick K. Li, Jerome Boursier, Sonja Lang, Münevver Demir, Anna Mae Diehl, Andrew S. Allegretti, Bernd Schnabl, Raymond T. Chung, A. Sloan Devlin

**Affiliations:** a Department of Biological Chemistry and Molecular Pharmacology, Harvard Medical School, Boston, MA, USA; b Liver Center, Massachusetts General Hospital, Harvard Medical School, Boston, MA, USA; c Department of Medicine, University of California San Diego, La Jolla, CA, USA; d Department of Medicine, VA San Diego Healthcare System, San Diego, CA, USA; e Division of Pediatric Gastroenterology & Nutrition, Department of Pediatrics, Massachusetts General Hospital, Boston, MA, USA; f Department of Biochemistry, University of Wisconsin, Madison, USA; g Section of Digestive Diseases, Department of Medicine, Yale School of Medicine, New Haven, CT, USA; h Service d'Hépato-Gastroentérologie, Centre Hospitalier Universitaire d'Angers, Angers, France; i Laboratoire HIFIH UPRES EA3859, Université d'Angers, Angers, France; j University of Cologne, Faculty of Medicine, and University Hospital Cologne, Department of Gastroenterology and Hepatology, Cologne, Germany; k Department of Hepatology and Gastroenterology, Campus Virchow Clinic and Campus Charité Mitte, Charité Universitaetsmedizin Berlin, Berlin, Germany; l Division of Gastroenterology, Department of Medicine, Duke University Medical Center, Durham, NC, USA; m Division of Nephrology, Department of Medicine, Massachusetts General Hospital, Boston, MA, USA

**Keywords:** Gut bacteria, bile salt hydrolase, MASLD

## Abstract

Metabolic dysfunction-associated steatotic liver disease (MASLD) is the most prevalent chronic liver disease in the United States, yet therapeutic options remain limited. Emerging evidence implicates the gut‒liver axis and intestinal permeability in disease pathogenesis. Previous studies in animal models and human cell culture indicated that bile salt hydrolases (BSHs), which are gut bacterial enzymes that deconjugate host-derived bile acids, damage intestinal barrier integrity and cause liver damage through the generation of unconjugated bile acids (UBAs). However, the relevance of these findings to MASLD patients is unknown. Here, we demonstrate that BSH activity is elevated in fecal samples from MASLD patients with advanced liver fibrosis and correlates with reduced fecal bile acid levels, which is consistent with a proposed model of increased intestinal permeability during MASLD progression. Through anaerobic culturing and activity-guided screening, we identify diverse BSH-active bacteria from patient fecal samples, suggesting broad microbial contributions to bile acid deconjugation in MASLD patients. Importantly, small-molecule BSH inhibitors suppressed BSH activity in both fecal communities and monocultures from MASLD patients without affecting bacterial viability. These findings indicate that BSH activity is a microbial function associated with MASLD progression and suggest that BSH inhibitors could be developed as a microbiome-targeted strategy for MASLD treatment.

## Introduction

Metabolic dysfunction-associated steatotic liver disease (MASLD), formerly known as nonalcoholic fatty liver disease (NAFLD), is now the leading cause of chronic liver disease in the West. Currently, as many as one-third of American adults are thought to have MASLD.^1^ Metabolic dysfunction-associated steatohepatitis (MASH), the progressive form of MASLD, is characterized histologically by fat deposition, lobular inflammation, and hepatocellular ballooning, together with hepatic fibrosis, and often leads to cirrhosis and hepatocellular carcinoma.^2^^,^^3^

Current treatment options for these diseases include lifestyle interventions, medication, and surgery. While off-label use of type 2 diabetes (T2D) drugs such as PPAR*-*γ agonists and GLP-1 agonists has become common, evidence for benefits has been limited.^4^ Although many agents have undergone trials, most have failed, and there is currently only one approved medication, resmetirom,^5^ for moderately advanced MASH, which produces only limited benefit. Unfortunately, once advanced liver cirrhosis has developed, the only cure is liver transplant.^6^ Earlier intervention would not only treat MASLD but could also prevent the morbidity and mortality of cirrhosis, liver failure and liver cancer.^3^

The crosstalk between the liver and the gut—the gut‒liver axis—has emerged as a key area of focus for uncovering the mechanisms that drive the development and progression of MASLD/MASH. The homeostasis of the gut-liver axis is in large part regulated by the intestinal epithelium, which forms a dynamic, selectively permeable, and tightly sealed barrier.^7^ Under pathologic conditions, tight junction proteins can become disrupted, resulting in leakage of dietary and bacterial antigens directly inducing hepatic inflammation.^8^ Emerging data have implicated increased intestinal permeability as an early feature of MASLD contributing directly to the development of liver injury.^9-12^ A recent meta-analysis demonstrated that MASLD patients had a significantly increased prevalence of intestinal permeability compared to healthy controls.^9^ This intestinal barrier damage results in inflammatory factors translocating from the gut into the portal vein and reaching the liver, where they contribute to the pathogenesis of MASLD.^13^

Bile acid (BAs) have been implicated as potential causal agents in the development of pathological intestinal permeability^14^^,^^15^ and MASLD/MASH.^16^^,^^17^ Bile acids are synthesized from cholesterol in the liver and are present in high concentrations in the gut (~10 mM in the small intestine and ~1 mM in the colon).^18^ Host liver enzymes append taurine or glycine to bile acids forming conjugated bile acids (CBAs),^19^ which are then secreted into the small intestine postprandially. In the lower GI tract, bacterial bile salt hydrolase (BSH) enzymes hydrolyze the amide bonds of these compounds, resulting in unconjugated bile acids (UBAs).^20^ This deconjugation enables further bacterial metabolism to occur. Following enterohepatic recirculation, these molecules can be re-converted to conjugated primary and secondary bile acids in the liver and then re-secreted into the gut.^21^ BSH enzymes are widespread in human gut bacteria and have no mammalian homolog.^22^^,^^23^

In recent work, we found that the products of BSH activity (UBAs) causally contribute to pathogenic intestinal permeability, while conjugated bile acids (CBAs) protect against this damage.^19^ Exposure of epithelial monolayers of human intestinal cells to UBAs resulted in barrier damage, including disruption of tight junctions and cell death. Increased intestinal permeability was also observed as an early feature of liver disease in an animal model of MASLD, and inhibition of BSH activity protected against pathogenic intestinal permeability and liver damage *in vivo*.^19^ Using a small molecule inhibitor of bacterial BSHs,^24^^,^^25^ we showed that inhibition of BSH activity increased CBA abundance and prevented the development of increased pathogenic intestinal permeability, hepatic steatosis, and hepatic inflammation in rats fed a choline-deficient, L-amino acid-defined high-fat diet.^19^ Together, these data suggest that high levels of UBAs increase intestinal permeability while CBAs protect against epithelial damage *in vitro* in human cells and *in vivo* in rats. However, the relevance of these findings to human MASLD patients remains unclear. Here, we investigated the relationships between the gut microbiome community composition, BSH activity, and progression of MASLD in humans using patient samples. We found that fecal BSH activity increased and total fecal BA levels decreased as liver fibrosis worsened in MASLD patients, which is consistent with a putative model in which increased BSH activity results in increased pathogenic intestinal permeability and liver damage. We observed that increased BSH activity was not confined to one type of bacteria but rather that numerous BSH-high bacteria were culturable from MASLD feces. Finally, small molecule BSH inhibitors suppressed BSH activity in both MASLD fecal communities and in individual strains cultured from MASLD feces. These data motivate future work investigating whether BSH inhibitors could be developed as therapies for MASLD.

## Results

**Figure 1.**
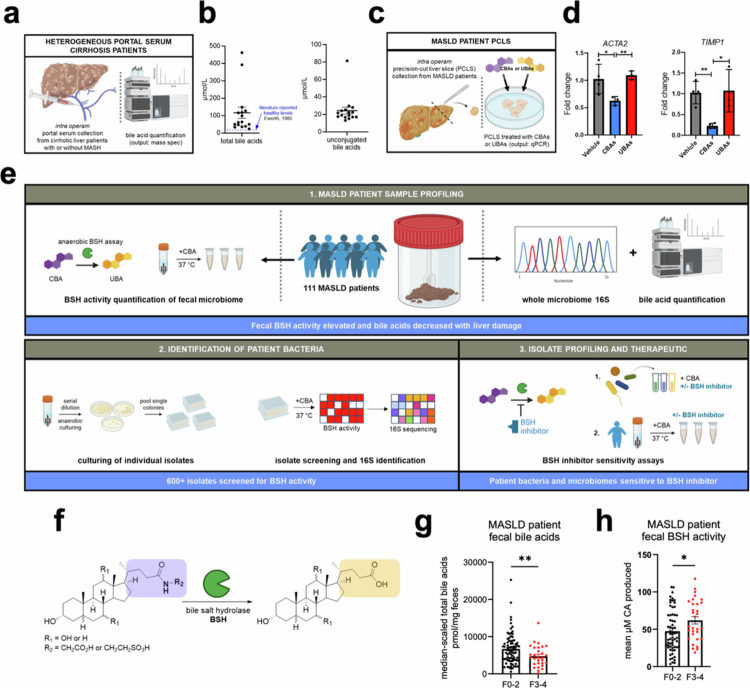
Bile salt hydrolase activity and bile acid levels correlate with liver damage. a) Portal serum was collected from patients with liver cirrhosis during surgery. b) Total bile acids were increased in the portal blood of cirrhotic liver patients (with or without MASH) compared to literature reports of nonliver disease patients (*n* = 16). Bile acids quantified by UPLC‒MS. c) Schematic overview of the precision-cut liver slice (PCLS) assay. d) Treatment of PCLS with CBAs (50 μM total) decreased expression of profibrogenic genes *ACTA2* and *TIMP1* compared to UBAs (50 μM total) by qPCR analysis. The CBA pool consisted of a 3:1 mixture of glyco-bile acids (GCA, GCDCA, GUDCA, GDCA, and GLCA in equal proportion) to tauro–bile acids (TCA, TCDCA, TUDCA, TDCA, and TLCA in equal proportions). UBA pool consisted of CA, CDCA, UDCA, DCA, and LCA in equal proportions (*n* = 3‒4 biological replicates per group, one-way ANOVA, experiments were repeated 3× with similar results). e) Graphical overview of the study design using 111 fecal samples from patients along the MASLD spectrum. Bile acids were quantified, and sample BSH activity was measured. Single isolates were cultured from representative high-BSH samples and screened for BSH activity. High-BSH isolates were sequenced and tested for sensitivity to a BSH inhibitor. f) Schematic of bacterial BSH activity, which converts conjugated bile acids (CBAs) to unconjugated bile acids (UBAs). g) Total fecal bile acid levels were decreased in patients with higher liver damage (*n* = 111). Bile acids quantified by UPLC‒MS. h) BSH activity in MASLD patient samples (*n* = 111) was increased in patients with higher fibrosis scores. Fecal samples were resuspended in anaerobic PBS and fed GCA, incubated for 2 h, and end point analyzed via UPLC‒MS. For each patient, BSH assays were performed in technical triplicate and the resultant mean was plotted as a single dot. For (g) and (h), two-tailed Welch's *t* tests were performed. *p < 0.05, **p < 0.01, ***p < 0.001, and ns = not significant. The data are presented as mean ± SEM.

The portal vein is the major conduit carrying blood from the gut to the liver. In our prior work, we showed that portal bile acid levels increased as liver damage progressed in an animal model of MASLD, reflecting increased pathogenic intestinal permeability.^19^ To investigate whether portal bile acid levels were also increased in patients with liver damage, portal serum samples were collected from 16 advanced cirrhotic liver patients with mixed etiologies (Table S1) undergoing transjugular intrahepatic portosystemic shunt (TIPS) placement as a treatment for portal hypertension and analyzed using ultra high-performance liquid chromatography‒mass spectrometry (UPLC‒MS) (Figure 1a and b). Owing to modern patient protection protocols, it was not possible to obtain portal blood from healthy subjects or MASLD-specific cohorts in early disease. As a result, we compared our values with levels reported in the older literature from patients with uncomplicated gallstone disease or undergoing elective cholecystectomy with normal liver function. We observed that total BA levels were substantially higher in portal blood from cirrhotic liver patients (range 24–462 µM, mean 116 µM) compared to reported values of patients without liver disease (mean 15 µM, Figure 1a and b, Tables S1, S2 and Figure S1). Notably, while UBA levels were low to undetectable in patients with normal liver function,^26-29^ portal UBAs were present in micromolar concentrations in advanced liver disease patients (mean 28 µM). These data suggest that epithelial barrier integrity is compromised in patients with advanced liver fibrosis.

Having previously demonstrated that UBAs damage intestinal integrity while CBAs protect against this damage *in vitro*, we sought to explore the effects of UBAs and CBAs on liver tissue (Figure 1c). We were able to obtain precision-cut liver slices (PCLSs) from a MASLD patient undergoing surgery. Treatment of these PCLSs with CBAs decreased the expression of the key profibrogenic genes actin alpha 2 (*ACTA2*)^30^ and tissue inhibitor of metalloproteinases 1 (*TIMP1*),^31^ whereas UBAs did not (Figures 1d and S2a). Similarly, treatment with CBAs reduced the expression of *ACTA2* and the fibrosis gene collagen type I alpha 1 chain (*COL1A1*) in primary human stellate cells (pHSCs) (Figure S2b). Treatment of the human hepatic stellate cell line LX2 with UBAs resulted in increased expression of *ACTA2* and *COL1A1* as well as the proinflammatory gene tumor necrosis factor-alpha (*TNFα*) (Figure S2c). Together, these findings indicate that UBAs induce the expression of profibrogenic genes in liver cells, while CBAs suppress profibrogenic gene induction. This work raised the question of whether bacterial BSH activity, which converts CBAs into damaging UBAs, is altered in MASLD patients (Figure 1e and f).

To investigate this question, we quantified fecal BA levels and BSH activity in 111 fecal samples from MASLD patients across a range of fibrosis stages (F0–F4) (Table S3). Total fecal bile acid from patients with advanced fibrosis (fibrosis stage 3 or 4; corresponding to advanced fibrosis and cirrhosis, respectively) were reduced compared to levels in earlier-stage patient samples (fibrosis score of 0–2) (Figures 1g and S3). This finding of decreased total fecal bile acids in later-stage disease is consistent with recent *in vivo* work.^19^ Next, we adapted an anaerobic BSH activity assay to measure bacterial BSH ability in frozen patient stool samples.^19^ Intriguingly, fecal BSH activity was higher in patients with more advanced liver fibrosis (Figure 1h). These data suggest a correlation between increased BSH activity, higher gastrointestinal permeability, and increased liver damage in MASLD patients.

**Figure 2.**
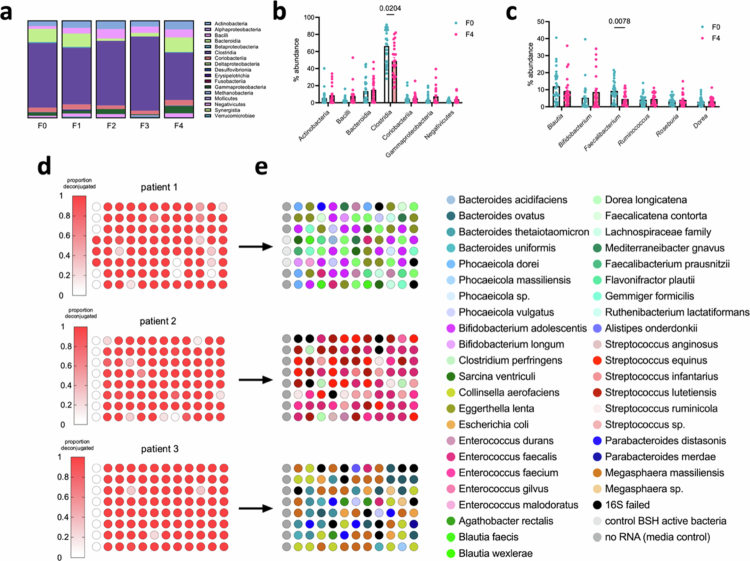
BSH activity-guided screening of cultured isolates identifies BSH-high bacterial isolates from 37 different species. a) 16S rRNA sequencing of each patient fecal sample was performed, and the results were grouped by fibrosis score (*n* = 111). No substantial shifts in class-level percent abundance were observed. b) At the class level, *Clostridia* were less abundant on average in patient fecal samples with F4 liver damage. c) Among the top ten most abundant genera, *Faecalibacterium* was the only bacterium whose abundance was significantly different between the F0 and F4 groups, and its levels were decreased. d) BSH activity from isolates of representative BSH-high patients 1–3. Individual isolates were isolated as described in Figure S4 and incubated with 50 µM GLCA and 50 µM GCA for 24 h. The resulting metabolites were analyzed by UPLC–MS. BSH screening assays were performed once in singlicate (one culture per isolate) given the high volume of isolates. Please refer to Table S4 for BSH activity and bacterial identity of each patient isolate tested. e) Corresponding 16S rRNA sequencing results from bacteria isolate plates in d. Aggregate data are summarized in Table S4. In (b) and (c), one-way ANOVA followed by Tukey's multiple comparisons test were performed. *p < 0.05, **p < 0.01, ***p < 0.001, and ns = not significant. The data are presented as mean ± SEM.

We next investigated whether any bacterial community members correlated with BSH activity in the patient samples. We performed 16S rRNA microbial community sequencing on all 111 fecal samples. Surprisingly, no compelling correlations between the degree of hepatic fibrosis and the abundance of specific genera or classes were found (Figure 2a and c). When comparing the extreme ends of the fibrosis spectrum (F0 and F4) at the class level, the abundance of *Clostridia* was significantly lower in F4 patients (Figure 2b). Among the top 10 most abundant genera in the samples, only *Faecalibacterium* was found to be statistically different between F0 and F4 cohorts, and its levels were decreased in F4 patients (Figure 2c). We did not observe any bacterial genera with significantly increased abundance that could have accounted for the increased BSH activity.

**Figure 3.**
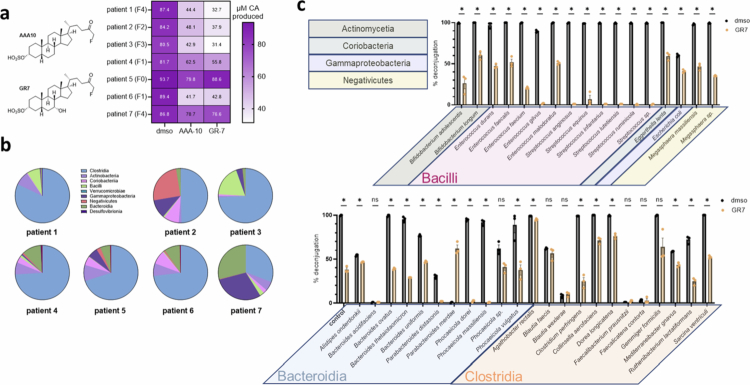
BSH inhibitors suppress unconjugated bile acid production in a panel of MASLD bacterial isolates. a) Two previously developed BSH inhibitors, AAA-10 and GR-7, inhibited BSH activity in seven representative microbiome communities from BSH-high patient feces. Fecal samples were resuspended in anaerobic PBS, incubated with 100 µM GCA ± 100 µM BSH inhibitor for 2 h, and then analyzed via UPLC‒MS (lower values indicate less deconjugation, *n* = 3 biological replicates per group; because additional freeze‒thaw cycles can alter microbial viability, assays were completed in a single experimental run). b) Class-level analysis of 16S rRNA sequencing data from the representative patient samples used in (a). c) GR-7 inhibited BSH activity in a wide array of BSH-containing bacteria from MASLD patient feces. Isolates were cultured in media containing 50 µM each of GLCA and GCA and either vehicle, 100 µM of AAA-10, or 100 µM of GR-7 and incubated at 37 °C for 24 h. Resultant metabolites were extracted for UPLC‒MS analysis (*n* = 3 biological replicates per group, and the experiments were repeated at least twice with similar results). In (c), two-tailed Welch's *t* tests were performed. *p < 0.05, **p < 0.01, ***p < 0.001, and ns = not significant. The data are presented as mean ± SEM.

Because our major finding was that BSH activity, not bacterial community composition, differed between patient groups, we pivoted and sought to isolate bacteria directly from MASLD patient feces and test their BSH activity. We developed an activity-guided workflow to isolate and then screen individual bacterial strains for their BSH activity (Figure S5a). Patient samples were anaerobically resuspended, diluted, and plated on supplemented brain–heart infusion (BHI+) media, which has been previously shown to promote the growth of diverse gut microbes.^32-34^ Individual isolates were restreaked to confirm monocolonization and then moved into a 96-well liquid growth format. From three representative fecal samples from F2–F4 groups with high BSH activity (patients 1–3 in Figure 3a), we cultured 616 isolates, which we then screened for BSH activity in monocultures (Figures 2d and S4). We then performed 16S rRNA sequencing (Sanger method) on the plate with the highest overall BSH activity per patient sample to determine bacterial identity (264 total isolates, Figure 2d,e and Table S4). This sequencing revealed that we cultured isolates from most of the major genera found in the human gut (e.g. *Blautia, Bacteroides, Bifidobacterium, Clostridium*, and *Faecalibacterium*).^35^ Among the sequenced isolates, the majority of the species isolated (90%) exhibited BSH activity *in vitro*. In sum, 41 unique species belonging to 22 unique genera were identified, 37 of which possessed BSH activity. These results indicate that representative MASLD patient samples contain a diverse array of culturable bacteria with BSH activity. More broadly, the isolate and whole microbiome sequencing and isolate culturing results highlight the ubiquitous nature of BSH activity across many genera and classes.

Having identified a positive correlation between more advanced fibrosis stages and BSH activity, we next wanted to investigate whether small molecule BSH inhibitors we had previously used *in vivo* could inhibit BSH activity in MASLD patient microbiomes.^19^^,^^24^^,^^25^ A subset of seven patient fecal samples with high BSH activity were anaerobically resuspended and incubated with a CBA in the presence of two previously published BSH inhibitors, AAA-10 and GR-7 (Figure 3a).^24^^,^^25^ Inhibitor treatment suppressed BSH activity of all but one patient sample, with GR-7 outperforming AAA-10. The microbial composition of these samples was diverse, suggesting that BSH inhibitors could suppress BSH activity in a variety of different gut bacteria (Figure 3b). To further investigate the sensitivity of BSH-containing bacteria on individual isolates, we tested the effects of GR-7, the most effective inhibitor in the community setting, on individual disease-associated bacteria in monoculture. GR-7 inhibited BSH activity in the vast majority of the isolates tested (29 of 37 monocultures had at least 30% suppression of BSH activity). However, some bacteria were insensitive to GR-7, including the Gram-positive strains *Agathobacter rectalis*, *Blautia faecis*, and *Blautia wexlerae* (Figure 3c). Importantly, GR-7 did not affect bacterial viability in any of the cultured isolates tested, indicating that the BSH inhibitory effects observed were not due to growth inhibition (Figure S5). Taken together, these data demonstrate that GR-7 can suppress BSH activity in both *ex vivo* MASLD communities and individual MASLD-associated cultured bacterial isolates. Further, this work suggests that in future studies, BSH inhibitors could be investigated as potential therapeutic agents for the prevention or treatment of MASLD.

## Discussion

In prior work, we showed that UBAs damage intact epithelial barriers both in human cells and in a rat model of MASLD, CBAs protect against this damage, and the use of a BSH inhibitor increases CBA levels and prevents pathogenic intestinal permeability and liver damage *in vivo*.^19^ Here, we extended our studies to investigate the potential relevance of our findings in patients. We showed that there are substantially higher levels of bile acids in the portal serum of patients with advanced cirrhosis (with or without MASH) compared to patients with normal liver function and that UBA treatment increased the expression of the profibrogenic gene *ACTA2* in LX2 cells, pHSC cells, and MASLD patient PCLS tissues compared to CBA treatment. These results provide proof-of-concept that UBAs can enter portal circulation during disease and induce profibrogenic gene expression in human livers.

These studies motivated us to investigate the correlation between fecal BA levels and BSH activity and fibrosis stage in MASLD patients. By investigating 111 fecal samples from MASLD patients with a range of liver fibrosis (F0–F4), we found that fecal BA levels decreased and BSH activity increased with more advanced fibrosis. These results are consistent with our previous findings in rats with increased BSH activity and intestinal permeability with disease progression and indicate a positive correlation between BSH activity and liver fibrosis in human MASLD patients. 16S rRNA sequencing of patient samples did not reveal a dominant class or genus of bacteria that correlated with BSH activity; instead, by culturing bacteria from three representative BSH-high fecal samples, we found that strains from a diverse array of 19 different genera and 37 different species contained BSH activity. These results are consistent with prior literature reports, which have found BSH activity across a wide variety of Gram-positive and Gram-negative gut commensal species.^22^^,^^36^ These findings highlight the importance of progressing beyond microbial sequencing and instead testing bacterial metabolic activity to characterize bacterial functions and to reveal key microbiome associations in human patient data.

**Figure 4.**
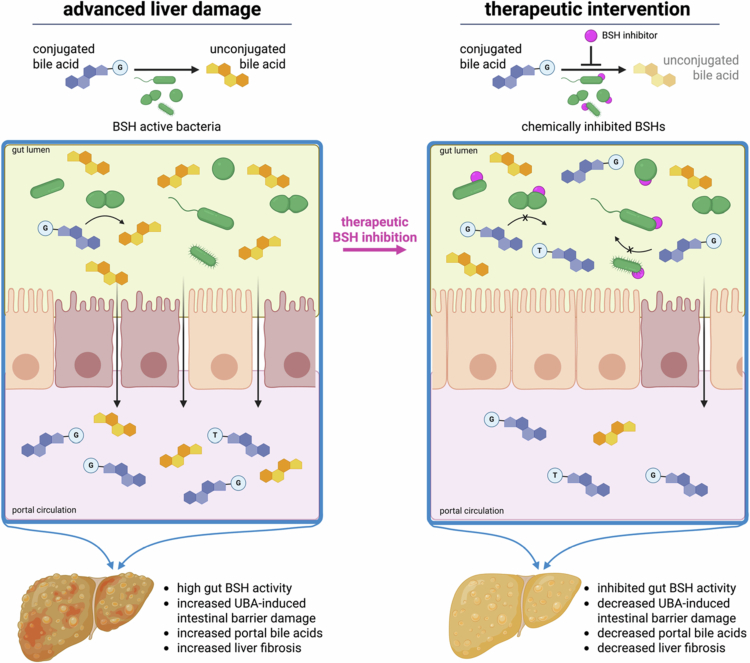
Proposed model for BSH inhibition as a potential therapy for MASLD. In MASLD, higher BSH activity in the gut microbiome produces higher levels of UBAs, which contribute to the development of pathogenic intestinal permeability. Epithelial barrier damage results in higher levels of bile acids in the portal vein. When these metabolites reach the liver, they contribute to liver fibrosis development. With the intervention of a BSH inhibitor, the production of damaging UBAs would be decreased, allowing the intestinal barrier to recover and reducing the levels of UBAs reaching the liver, thus reducing liver damage.

Finally, we showed that a BSH inhibitor we previously developed, GR-7,^24^ inhibits BSH activity in both *ex vivo* microbial communities from MASLD patients and individual isolates cultured directly from these communities. These results provide promising proof-of-concept that BSH inhibitors can limit UBA production by MASLD-associated human gut bacteria and suggest that BSH inhibitors could be developed as treatments for MASLD (Figure 4). Future work could build on these findings and explore therapeutic interventions with a BSH inhibitor prior to the establishment of irreversible liver damage, which we theorize could result in decreased UBA production by gut bacteria, reduced intestinal barrier damage, and decreased liver fibrosis.

Importantly, in addition to acting as detergents, bile acids also signal through host receptors, including the farnesoid X receptor or FXR, a nuclear hormone receptor, and the G protein-coupled receptors GPBAR1/TGR5 and the sphingosine-1-phosphate receptor 2 or S1PR2.^37^ Prior work shows that these pathways can influence intestinal permeability, with effects that vary by bile acid conjugation state and tissue context. For example, secondary bile acids that activate TGR5 promote GLP-1 release and have been implicated in preserving barrier integrity.^38^ FXR signaling also modulates junctional proteins and inflammation, but reports differ across models—some studies find FXR activation supports barrier function, whereas others have reported that FXR deletion can attenuate barrier dysfunction in acute colitis settings.^39^^,^^40^ Certain CBAs can activate S1PR2 more effectively than their UBA counterparts, and S1PR2 has been linked to barrier regulation, although the results have been mixed. S1PR2 activation has been reported to strengthen tight junctions via PI3K/AKT in epithelial cells, while in inflammatory contexts, S1PR2 expression increases and its inhibition reduces permeability and injury in DSS colitis models.^41^^,^^42^ In our MASLD animal model, we observed no gross differences in FXR or TGR5 signaling between groups, and epidermal growth factor receptor (EGFR) activation in Caco-2 cells exposed to bile acid pools was also similar.^19^ Taken together with increased BSH activity and established permeability, our data support a primarily physicochemical mechanism for gut epithelial barrier injury in our system.

We also note that bile acid signaling may be relevant in the liver:^43^^,^^44^ our proof-of-concept studies with PCLS, LX2 cells, and pHSCs show that CBAs and UBAs differentially affect profibrogenic gene expression, which is consistent with the idea that the conjugation state can shift the fibrogenic status of liver cells. We did not determine the mechanism in those assays—differences could reflect physicochemical or receptor-mediated effects—and future work should delineate the signaling pathways that mediate CBA- vs UBA-dependent responses in hepatic cells. Accordingly, our therapeutic rationale is not to ablate all secondary bile acid signaling but to reduce UBA-driven barrier damage by shifting the pool toward CBAs via BSH inhibition, while recognizing the beneficial roles of secondary bile acids in gut homeostasis.

### Future opportunities in diagnostics

Importantly, these studies also provide the initial stages of a potential roadmap for screening and treatment of MASLD patients for their responsiveness to BSH inhibitor treatment. Gut microbial sequencing of patient feces is time-consuming, and our data suggest that the results are unlikely to reveal whether the microbiome of a patient will be sensitive to a BSH inhibitor. In contrast, fecal BSH activity profiling could, in theory, provide a rapid answer as to whether a patient would respond to a particular BSH therapy, that is, whether this therapy would be likely to block UBA production *in vivo*. Such a theoretical protocol might proceed as follows: within three hours of receipt of a patient's frozen fecal sample (a 2-h incubation assay followed by 1 h for mass spectrometry analysis), a clinical chemistry laboratory with a standard single quadrupole mass spectrometer could determine both the BSH activity of a given patient and the ability of a set of BSH inhibitors to suppress this BSH activity.

There is growing recognition that personalized medicine tailored to individual patients has the potential to optimize the standard of care, particularly for a heterogeneous disorder such as MASLD.^45-47^ Ordinarily, such efforts are based on a patient's genomic profile. Here, we propose that the functional properties of a patient's microbiome could be used to guide their treatment plan. While substantial further work would be needed to make such care a reality, we believe this work provides ample rationale for these studies and makes a case for activity-based profiling over sequencing-based microbiome analysis.

### Limitations of this work

Fecal sampling was performed over a period of years, and while best efforts were taken to preserve the integrity of the samples, some fecal bacteria likely died between sample collection and our culturing studies. Culturing and isolation to identify individual bacteria was performed using a single rich media, which could have prevented the growth and identification of some bacteria. Information about diabetes status, BMI, or medications was not available for the fecal cohorts, limiting our ability to evaluate potential confounding effects. Likewise, we obtained a heterogeneous group of portal serum samples from cirrhotic liver patients with mixed etiologies, not a MASLD/MASH-specific cohort. While the fibrosis score and degree of liver scarring for each patient was known for the fecal samples, the intestinal permeability of the patient was not measured directly and was inferred based on the collected sample data. Moreover, we did not have access to paired patient blood samples to investigate metabolite levels in systemic circulation.

GBAs were used for BSH activity assays because they are the predominant conjugated BA form in humans.^48^ We did not include tauro-conjugated bile acids (TBAs) because they constitute a smaller proportion of the human bile acid pool, and the screening nature of this work necessitated that a limited number of substrates be used to facilitate downstream analysis. Finally, this work focused on the relationships among gut microbial function, BSH activity, and fibrosis progression in MASLD patients. It is possible that other microbial activities affect epithelial integrity and liver function in this patient population. Despite these limitations, this work illustrates a link between microbial bile acid deconjugation and the pathogenesis of MASLD/MASH in human patients.

### Conclusion

Taken together with prior work in human intestinal cells and an animal model of MASLD, our data suggest that BSH activity is a novel target for the prevention or treatment of liver diseases characterized by pathogenic intestinal permeability, including MASLD.

## Materials and methods

### Reagents

All bile acids and reagents for synthesis were commercially purchased from Steraloids Inc. and Sigma-Aldrich. Stock solutions of all bile acids and inhibitors were prepared in molecular biology grade DMSO (Sigma-Aldrich) at 1000× concentrations. The solvents used for preparing UPLC‒MS samples were HPLC grade.

### Human samples

For human portal vein serum collection, adult nonpregnant patients (age > 18 y) with decompensated cirrhosis referred to MGH interventional radiology for transjugular intrahepatic portosystemic shunt (TIPS) placement provided serum taken directly from the portal vein at the time of TIPS placement. Patients provided written informed consent under Partners IRB 2015P001285. MASLD human liver samples were obtained in accordance with the protocol approved by the Mass General Brigham Institutional Review Board (IRB), protocol #1999P004983. Human stool samples were obtained from individuals with metabolic dysfunction-associated steatotic liver disease (MASLD) under protocols approved by institutional review boards at the respective collection sites (Cologne University's Faculty of Medicine, reference #15-056, UC San Diego IRB #171246XX, and Duke IRB #Pro00007550). All the stool samples were stored at –80 °C immediately after collection and processed using identical protocols in the Devlin laboratory. A subset of samples used in this study has been previously described.^49^

### Bile acid quantification

Bile acid profiling by UPLC–MS was performed using a published method.^36^ Patient serum samples were removed from −80 °C storage and thawed. The samples were diluted 1:1 with methanol and then centrifuged at maximum speed for 10 min. The samples were frozen until further analysis. Patient fecal samples were removed from −80 °C storage and kept on dry ice. The samples were placed on a sterile Petri dish, and a portion of the sample was excised and placed in a tared homogenizing tube with ceramic beads. The tissues were homogenized at maximum speed for two intervals of 30 s, then centrifuged at maximum speed for 10 min and frozen until further analysis. For both tissue types, a portion of the thawed supernatant was diluted 1:1 with 50% methanol in water and again centrifuged at maximum speed before UPLC‒MS injection and analysis. The limits of detection and chromatographic specification for individual bile acids have been previously described.^36^ Correction factors for extraction efficiency were used and were determined by extraction of known concentrations of relevant bile acids from buffer or bacterial media and comparison to standard curves. Fecal bile acids were analyzed in three batches, and batch effects were corrected for with median-scaling for individual BA levels (e.g. batches 2 and 3 were scaled to the median of batch 1).

### *Ex vivo* treatment of PCLS with bile acids

PCLS were performed as previously described.^50^^,^^51^ PCLS were obtained from the nontumor liver of a 59-y-old man with MASLD who underwent hepatic resection for circumscribed colorectal cancer metastasis (steatosis without fibrosis were seen on histology).The MASLD liver tissue was cored using a 10-mm biopsy punch (Acuderm Inc.), superglued to the vibratome mounting stage, submerged in a media chamber containing sterile Krebs Henseleit buffer (Sigma-Aldrich) and cut using a 7000smz-2 Vibratome (Campden Instruments Limited) with a 7550-1-C ceramic blade (Campden Instruments Limited) at an advanced speed of 0.1 mm/s, 2.5 mm amplitude, 50 Hz frequency and a thickness of 250 μm. PCLS were transferred onto 8-μm-pore Transwell inserts and cultured in standard 6-well plates (Corning) on a rocking platform (Boekel Scientific). All PCLS were cultured in William's E medium (Sigma-Aldrich) containing 2.0 g/L glucose, 15% fetal bovine serum, 2 mM L-glutamine supplement, 100 U/mL penicillin, and 100 μg/mL streptomycin and further treated with a pool of CBAs (50 μM total; 7.5 μM each of glycocholic acid, glycochenodeoxycholic acid, glycourosodeoxycholic acid, glycodeoxycholic acid, glycolithocholic acid, and 2.5 μM each of taurocholic acid, taurochenodeoxycholic acid, taurourosodeoxycholic acid, taurodeoxycholic acid, and taurolithocholic acid), a pool of UBAs (50 μM total; 10 μM each of cholic acid, chenodeoxycholic acid, urosodeoxycholic acid, deoxycholic acid, and lithocholic acid), or DMSO for 48 h at 37 °C in a humidified atmosphere of 5% CO_2_. We used 50 μM for both UBA and CBA treatments because this concentration fell between the mean portal serum concentrations of UBAs (~25 μM) and CBAs (~80 μM).

### Treatment of LX2 cells with bile acids

LX2 cells were obtained from Dr Scott Friedman at the Icahn School of Medicine at Mount Sinai. The cells were grown in Dulbecco's modified Eagle's medium (DMEM, Cellgro) containing 15% fetal bovine serum (Gibco), 1 mM sodium pyruvate (Gibco), 100 U/mL penicillin sodium, and 100 μg/mL streptomycin sulfate (Lonza) and further treated with a pool of 3:1 glyco- to tauro-CBAs (250 μM total; 37.5 μM each of glycocholic acid, glycochenodeoxycholic acid, glycourosodeoxycholic acid, glycodeoxycholic acid, and glycolithocholic acid, and 12.5 μM each of taurocholic acid, taurochenodeoxycholic acid, taurourosodeoxycholic acid, taurodeoxycholic acid, and taurolithocholic acid), a pool of UBAs (250 μM total; 50 μM each of cholic acid, chenodeoxycholic acid, urosodeoxycholic acid, deoxycholic acid, and lithocholic acid), or DMSO for 48 h at 37 °C in a humidified atmosphere of 5% CO_2_. We selected 250 μM for both UBA and CBA treatments because this concentration fell within the range of total BAs detected in human portal serum samples and was within an order of magnitude of the mean total BA concentration (Figure 1b).

### Treatment of pHSCs with bile acids

pHSCs were obtained through the Liver Tissue and Cell Distribution System (LTCDS) at Department of Surgery, University of Pittsburgh. The cells were cultured in DMEM (Cellgro) supplemented with 15% fetal bovine serum (Gibco), 0.4% L‐glutamine (Gibco), 100 U/mL penicillin sodium, and 100 μg/mL streptomycin sulfate (Lonza) and further treated with a pool of 3:1 glyco- to tauro-CBAs (250 μM total; 37.5 μM each of glycocholic acid, glycochenodeoxycholic acid, glycourosodeoxycholic acid, glycodeoxycholic acid, and glycolithocholic acid, and 12.5 μM each of taurocholic acid, taurochenodeoxycholic acid, taurourosodeoxycholic acid, taurodeoxycholic acid, and taurolithocholic acid), a pool of UBAs (250 μM total; 50 μM each of cholic acid, chenodeoxycholic acid, urosodeoxycholic acid, deoxycholic acid, and lithocholic acid), or DMSO for 48 h at 37 °C in a humidified atmosphere of 5% CO_2_. We selected 250 μM for both UBA and CBA treatments because this concentration fell within the range of total BAs detected in human portal serum samples and was within an order of magnitude of the mean total BA concentration (Figure 1b).

### Gene expression analysis by RT-qPCR

Total RNA was isolated from cells or PCLS using TRIzol (Invitrogen) and subsequently treated with DNase I (Promega). For reverse transcription (RT), one microgram of total RNA from each sample was used to synthesize cDNA with a high-capacity cDNA reverse transcription kit (Thermo Fisher). Quantitative real-time polymerase chain reaction (qPCR) was performed with POWRUP SYBR master mix (Thermo Fisher) on the 7900HT Fast Real-Time PCR System (Thermo Fisher) platform. The 2^−ΔΔCt^ method was used to calculate relative changes in gene expression. Primer sequences were obtained from https://www.ncbi.nlm.nih.gov/tools/primer-blast/index.cgi (as shown in Supplemental Table 5), and primers were purchased from Invitrogen.

### BSH activity assay of human feces

Patient fecal samples were removed from −80 °C storage and kept on dry ice. The samples were placed on a sterile Petri dish. A non-air-exposed portion of the sample (approximately 20–50 mg) was excised, placed in a premassed conical tube, and then weighed. The samples were brought into an anaerobic chamber, and a fecal slurry with anaerobicized PBS was made at a concentration of 5 mg wet mass per mL. The samples were vortexed gently, and in triplicate 100 µL of the slurry was diluted into 400 µL of PBS containing glyco cholic acid (GCA). Final assay concentrations were 1 mg/mL feces and 100 µM GCA. The samples were incubated for 2 h and then quenched with 500 µL of methanol containing a deuterated internal standard. The samples were frozen until mass spectrometry analysis, after which they were thawed, vortex, and spun down before UPLC‒MS injection and analysis. % deconjugation was calculated as [concentration of CA]/[concentration of CA + GCA] × 100. Background samples of 1 mg/mL feces with no bile acid added were also analyzed to ensure no interfering background levels of endogenous bile acids.

### Bacterial culturing

All bacterial strains were cultured at 37 °C in BHI+ media, which consists of brain heart infusion (BHI, Bacto) supplemented with 1% BBL vitamin K1-hemin solution (BD), 1% trace mineral solution (ATCC), 1% trace vitamins (ATCC), 5% heat-inactivated fetal bovine serum (FBS, HyClone), 1 g/L cellobiose, 1 g/L maltose, 1 g/L fructose, and 0.5 g/L L-cysteine. BHI is a rich media that supports the growth of a diverse array of anaerobic bacteria, and the additives further support the growth of fastidious microorganisms from the mammalian gut.^24^^,^^32^^,^^34^ All strains were grown under anaerobic conditions in an anaerobic chamber (Coy Lab Products Airlock) with a gas mix of 5% hydrogen and 20% carbon dioxide nitrogen.

### 16S rRNA sequencing of whole microbiome communities

Frozen fecal samples were minimally thawed, and ~200 µL was taken for extraction. DNA extraction was performed with the ZymoBIOMICS™ DNA Miniprep Kit5 following Appendix B for solid masses. The variable region 4 of the 16S rRNA genes was amplified using primers: forward, 5′-CCTAYGGGNBGCWGCAG-3′; reverse, 5′-GACTACNVGGGTMTCTAATCC-3′. The quality of the amplified DNA products was checked, and an aggregated library was generated for Illumina MiSeq sequencing. Demultiplexed FASTQ files were generated by the Illumina MiSeq software using default parameters, and quality control was done by the pipeline at Azenta Life Sciences. The phylogenetic affiliation of each OTUs were aligned to the NCBI database and assessed based on percent identification > 98%.

### 16S rRNA sequencing (Sanger method) of individual isolates

Isolates were grown in BHI+ media anaerobically overnight. 500 µL of culture was centrifuged at 12,500 RPM for 10 min, and the supernatant decanted away. Cell pellets were enzymatically purified as previously described.^24^ The variable region 4 of the 16S rRNA genes was amplified using primers: forward, 5′-TATGGTAATTGTGTGCCAGCMGCCGCGGTAA-3′; reverse, 5′-AGTCAGTCAGCC GGACTACHVGGGTWTCTAAT-3′. The quality of the amplified DNA products was checked, and roughly 120 ng of each DNA product was pooled together to generate an aggregated library for Illumina MiSeq sequencing. Demultiplexed FASTQ files were generated by the Illumina MiSeq software using default parameters, and quality control was done by the pipeline at SeqCenter. The phylogenetic affiliation of each OTUs were aligned to the NCBI database and assessed based on percent identification >98%.

### Activity-guided identification culturing and workflow

#### Isolate picking

Patient fecal samples were removed from −80 °C storage and kept on dry ice. The samples were placed on a sterile Petri dish, and a non-air-exposed portion of the sample was excised and placed in a conical tube to weight. Samples were brought into an anaerobic chamber, and a fecal slurry with anaerobicized PBS was made at a concentration of 5 mg wet mass per mL. The samples were vortexed gently and then serially diluted in anaerobicized PBS. Neat fecal slurry through 10−8 dilutions were plated by pipetting 100 µL of dilution onto a BHI+ agar plate and then homogenously spread with an L-shaped spreader. The plates were incubated anaerobically for 2 d, then individual colonies were picked and restreaked onto fresh BHI+ agar plates and incubated for an additional 3 d.

#### Activity screening and identification

In an anaerobic chamber, pure individual colonies were then transferred to 96-well plates containing 600 µL of BHI+ media; column 1 was reserved in each plate for media controls and 3 wells of known bacteria with BSH activity (*Bacteroides fragilis* ATCC 25285). The plates were incubated for 2 d to allow the colonies to reach the stationary phase (dilutions less than 10−4 usually grew lawns and were unusable). Saturated cultures were diluted 1:10 in fresh BHI+ media containing GCA and GLCA (final concentrations of 50 µM each) and incubated overnight. The cultures were then removed from the anaerobic chamber and acidified with 50 µL of 3 M sulfuric acid. Liquid‒liquid extraction was performed twice with ethyl acetate, and the combined organic layers were dried down with compressed gas. The extracts were redissolved in 1:3 water/methanol, centrifuged and analyzed by UPLC–MS. % deconjugation was calculated as [concentration of CA + LCA]/[concentration of CA + LCA + GCA + GLCA] × 100. Plates of isolates with high average BSH activity were sent for 16S sequencing.

#### BSH inhibitor sensitivity assay in human feces​​​​​

Patient fecal samples were removed from −80 °C storage and kept on dry ice. The samples were placed on a sterile Petri dish. A non-air-exposed portion of the sample (approximately 20–50 mg) was excised, placed in a premassed conical tube, and then weighed. The samples were brought into an anaerobic chamber, and a fecal slurry with anaerobicized PBS was made at a concentration of 5 mg wet mass per mL. The samples were vortexed gently, and in triplicate, 100 µL of the slurry was diluted into 400 µL of PBS containing BSH inhibitor GR-7 (the final concentrations were 1 mg/mL feces and 100 µM inhibitor). The inhibitor and fecal slurry were preincubated for 30 min (inverting samples every 10 min to ensure mixing) before GCA was added (100 µM). The samples were incubated for 2 h and then quenched with 500 µL of methanol containing a deuterated internal standard. The samples were frozen until mass spectrometry analysis, where they were thawed, vortex, and spun down before UPLC‒MS injection and analysis. % deconjugation was calculated as [concentration of CA]/[concentration of CA + GCA] × 100. Background samples of 1 mg/mL feces with no bile acid added were also analyzed to ensure that there were no interfering background levels of endogenous bile acids.

#### BSH inhibitor sensitivity assay in bacterial monocultures

Bacteria of interest were anaerobically grown on BHI+ agar plates over 2 d, and single colonies were used to inoculate 600 µL BHI+ media in triplicate in 96-well plates. After 2 d, saturated cultures were diluted 1:10 in fresh BHI+ media containing DMSO, AAA-10, or GR-7 (triplicate each condition, 100 µM inhibitor). A 1:1 mixture of GCA and GLCA were added to all conditions (100 µM), and the cultures were incubated overnight. After incubation, the plates were removed from the anaerobic chamber, and the cultures were acidified with 50 µL of 3 M sulfuric acid. Liquid‒liquid extraction was performed twice with ethyl acetate, and the combined organic layers were dried down with compressed gas. The extracts were redissolved in 1:3 water/methanol, centrifuged and analyzed by UPLC–MS. % deconjugation was calculated as follows: [concentration of CA + LCA]/[concentration of CA + LCA + GCA + GLCA] × 100.

## Supplementary Material

Supplementary material Supplementary Material.

Supplementary material Supplementary Material.

## Data Availability

16S sequencing data for MASLD patient fecal samples has been submitted to NCBI, SRA: PRJNA540738^52^ and PRJNA1294521. All other data are contained within the manuscript and supporting information.

## References

[1] Loomba R, Sanyal AJ. The global NAFLD epidemic. Nat Rev Gastroenterol Hepatol. 2013;10(11):686–690. doi:10.1038/nrgastro.2013.171. 24042449

[2] Younossi ZM, Koenig AB, Abdelatif D, Fazel Y, Henry L, Wymer M. Global epidemiology of nonalcoholic fatty liver disease—meta-analytic assessment of prevalence, incidence, and outcomes. Hepatology. 2016;64(1):73–84. doi:10.1002/hep.28431. 26707365

[3] Adams LA, Sanderson S, Lindor KD, Angulo P. The histological course of nonalcoholic fatty liver disease: a longitudinal study of 103 patients with sequential liver biopsies. J Hepatol. 2005;42(1):132–138. doi:10.1016/j.jhep.2004.09.012. 15629518

[4] Sanyal AJ, Chalasani N, Kowdley KV, McCullough A, Diehl AM, Bass NM, Neuschwander-Tetri BA, Lavine JE, Tonascia J, Unalp A, et al. Pioglitazone, vitamin E, or placebo for nonalcoholic steatohepatitis. N Engl J Med. 2010;362(18):1675–1685. doi:10.1056/nejmoa0907929. 20427778 PMC2928471

[5] Raja A, Subhash Sagar R, Saeed S, Zia ul haq A, Khan O, Dileep Bhimani P, Raja S, Deepak F, Ahmed M, Ashir Shafique M, et al. Safety and efficacy of resmetirom in the treatment of patients with non-alcoholic steatohepatitis and liver fibrosis: a systematic review and meta-analysis. Ann Med Surg. 2024;86(7):4130–4138. doi:10.1097/ms9.0000000000002195. PMC11230798 38989228

[6] Scaglione S, Kliethermes S, Cao G, Shoham D, Durazo R, Luke A, Volk ML. The epidemiology of cirrhosis in the united states a population-based study. J Clin Gastroenterol. 2015;49(8):690–696. doi:10.1097/MCG.0000000000000208. 25291348

[7] Seki E, De Minicis S, Österreicher CH, Kluwe J, Osawa Y, Brenner DA, Schwabe RF. TLR4 enhances TGF-β signaling and hepatic fibrosis. Nat Med. 2007;13(11):1324–1332. doi:10.1038/nm1663. 17952090

[8] Massier L, Blüher M, Kovacs P, Chakaroun RM. Impaired intestinal barrier and tissue bacteria: pathomechanisms for metabolic diseases. Front Endocrinol. 2021;12:616506. doi:10.3389/fendo.2021.616506. PMC7985551 33767669

[9] Luther J, Garber JJ, Khalili H, Dave M, Bale SS, Jindal R, Motola DL, Luther S, Bohr S, Jeoung SW, et al. Hepatic injury in nonalcoholic steatohepatitis contributes to altered intestinal permeability. Cell Mol Gastroenterol Hepatol. 2015;1(2):222–232. doi:10.1016/j.jcmgh.2015.01.001. 26405687 PMC4578658

[10] Miele L, Valenza V, La Torre G, Montalto M, Cammarota G, Ricci R, Mascianà R, Forgione A, Gabrieli ML, Perotti G, et al. Increased intestinal permeability and tight junction alterations in nonalcoholic fatty liver disease. Hepatology. 2009;49(6):1877–1887. doi:10.1002/hep.22848. 19291785

[11] Rahman K, Desai C, Iyer SS, Thorn NE, Kumar P, Liu Y, Smith T, Neish AS, Li H, Tan S, et al. Loss of junctional adhesion molecule a promotes severe steatohepatitis in mice on a diet high in saturated fat, fructose, and cholesterol. Gastroenterology. 2016;151(4):733–746.e12. doi:10.1053/j.gastro.2016.06.022. 27342212 PMC5037035

[12] Mouries J, Brescia P, Silvestri A, Spadoni I, Sorribas M, Wiest R, Mileti E, Galbiati M, Invernizzi P, Adorini L, et al. Microbiota-driven gut vascular barrier disruption is a prerequisite for non-alcoholic steatohepatitis development. J Hepatol. 2019;71(6):1216–1228. doi:10.1016/j.jhep.2019.08.005. 31419514 PMC6880766

[13] Benedé-Ubieto R, Cubero FJ, Nevzorova YA. Breaking the barriers: the role of gut homeostasis in metabolic-associated steatotic liver disease (MASLD). Gut Microbes. 2024;16(1):1–27. doi:10.1080/19490976.2024.2331460. PMC10962615 38512763

[14] Murakami Y, Tanabe S, Suzuki T. High-fat diet-induced intestinal hyperpermeability is associated with increased bile acids in the large intestine of mice. J Food Sci. 2016;81(1):H216–H222. doi:10.1111/1750-3841.13166. 26595891

[15] Papillon SC, Frey MR, Ford HR, Gayer CP. Secondary bile acids as a mechanism of intestinal injury. J Am Coll Surg. 2013;217(3):S13. doi:10.1016/j.jamcollsurg.2013.07.010.

[16] Lai J, Luo L, Zhou T, Feng X, Ye J, Zhong B. Alterations in circulating bile acids in metabolic dysfunction-associated steatotic liver disease: a systematic review and meta-analysis. Biomolecules. 2023;13(9):1356. doi:10.3390/biom13091356. 37759756 PMC10526305

[17] Gottlieb A, Canbay A. Why bile acids are so important in non-alcoholic fatty liver disease (NAFLD) progression. Cells. 2019;8:1358. doi:10.3390/cells8111358. 31671697 PMC6912605

[18] Hofmann AF. The continuing importance of bile acids in liver and intestinal disease. Arch Intern Med. 1999;159(22):2647–2658. doi:10.1001/archinte.159.22.2647. 10597755

[19] Li DK, Chaudhari SN, Lee Y, Sojoodi M, Adhikari AA, Zukerberg L, Shroff S, Barrett SC, Tanabe K, Chung RT, et al. Inhibition of microbial deconjugation of micellar bile acids protects against intestinal permeability and liver injury. Sci Adv. 2022;8(34):eabo2794. doi:10.1126/sciadv.abo2794. 36026454 PMC9417178

[20] Ridlon JM, Kang DJ, Hylemon PB. Bile salt biotransformations by human intestinal bacteria. J Lipid Res. 2006;47(2):241–259. doi:10.1194/jlr.R500013-JLR200. 16299351

[21] Peery AF, Crockett SD, Barritt AS, Dellon ES, Eluri S, Gangarosa LM, Jensen ET, Lund JL, Pasricha S, Runge T, et al. Burden of gastrointestinal, liver, and pancreatic diseases in the United States. Gastroenterology. 2015;149(7):1731–1741. doi:10.1053/j.gastro.2015.08.045.Burden. 26327134 PMC4663148

[22] Song Z, Cai Y, Lao X, Wang X, Lin X, Cui Y, Kalavagunta PK, Liao J, Jin L, Shang J, et al. Taxonomic profiling and populational patterns of bacterial bile salt hydrolase (BSH) genes based on worldwide human gut microbiome. Microbiome. 2019;7(1):1–16. doi:10.1186/s40168-019-0628-3. 30674356 PMC6345003

[23] Foley M, Flaherty SO, Barrangou R, Theriot CM. Bile salt hydrolases: gatekeepers of bile acid metabolism and host-microbiome crosstalk in the gastrointestinal tract. PLoS Pathog. 2019;15(3):e1007581. doi:10.1371/journal.ppat.1007581. 30845232 PMC6405046

[24] Adhikari AA, Seegar TCM, Ficarro SB, McCurry MD, Ramachandran D, Yao L, Chaudhari SN, Ndousse-Fetter S, Banks AS, Marto JA, et al. Development of a covalent inhibitor of gut bacterial bile salt hydrolases. Nat Chem Biol. 2020;16(3):318–326. doi:10.1038/s41589-020-0467-3. 32042200 PMC7036035

[25] Adhikari AA, Ramachandran D, Chaudhari SN, Powell CE, Li W, McCurry MD, Banks AS, Devlin AS. A gut-restricted lithocholic acid analog as an inhibitor of gut bacterial bile salt hydrolases. ACS Chem Biol. 2021;16(8):1401–1412. doi:10.1021/acschembio.1c00192. 34279901 PMC9013266

[26] Lindblad L, Lundholm K, Scherstén T. Bile acid concentrations in systemic and portal serum in presumably normal man and in cholestatic and cirrhotic conditions. Scand J Gastroenterol. 1977;12(4):395–400. doi:10.3109/00365527709181679. 560715

[27] Ahlberg J, Angelin B, Bjorkhem I, Einarsson K. Individual bile acids in portal venous and systemic blood serum of fasting man. Gastroenterology. 1977;73(6):1377–1382. doi:10.1016/s0016-5085(19)31517-3. 913977

[28] Einarsson K, Ahlberg J, Angelin B, Björkhem I, Ewerth S. Portal venous bile acids in cholesterol gallstone disease: effect of treatment with chenodeoxycholic and cholic acids. Hepatology. 1985;5(4):661–665. doi:10.1002/hep.1840050423. 4018738

[29] Linnet K, Andersen JR, Hesselfeldt P. Concentrations of glycine- and taurine-conjugated bile acids in portal and systemic venous serum in man. Scand J Gastroenterol. 1984;19(4):575–578. doi:10.1080/00365521.1984.12005772. 6463582

[30] Fuchs BC, Hoshida Y, Fujii T, Wei L, Yamada S, Lauwers GY, Mcginn CM, Deperalta DK, Chen X, Kuroda T, et al. Epidermal growth factor receptor inhibition attenuates liver fibrosis and development of hepatocellular carcinoma. Hepatology. 2014;59(4):1577–1590. doi:10.1002/hep.26898. 24677197 PMC4086837

[31] van Riet S, Julien A, Atanasov A, Nordling Å, Ingelman-Sundberg M. The role of sinusoidal endothelial cells and TIMP1 in the regulation of fibrosis in a novel human liver 3D NASH model. Hepatol Commun. 2024;8:e0374. doi:10.1097/hc9.0000000000000374. 38358377 PMC10871795

[32] Hall AB, Yassour M, Sauk J, Garner A, Jiang X, Arthur T, Lagoudas GK, Vatanen T, Fornelos N, Wilson R, et al. A novel ruminococcus gnavus clade enriched in inflammatory bowel disease patients. Genome Med. 2017;9(1):1–12. doi:10.1186/s13073-017-0490-5. 29183332 PMC5704459

[33] Chen Y, Chaudhari SN, Harris DA, Roberts CF, Moscalu A, Mathur V, Zhao L, Tavakkoli A, Devlin AS, Sheu EG. A small intestinal bile acid modulates the gut microbiome to improve host metabolic phenotypes following bariatric surgery. Cell Host Microbe. 2024;32(8):1315–1330.e5. doi:10.1016/j.chom.2024.06.014. 39043190 PMC11332993

[34] Paik D, Yao L, Zhang Y, Bae S, D'Agostino GD, Zhang M, Kim E, Franzosa EA, Avila-Pacheco J, Bisanz JE, et al. Human gut bacteria produce ΤΗ17-modulating bile acid metabolites. Nature. 2022;603:907–912. doi:10.1038/s41586-022-04480-z. 35296854 PMC9132548

[35] Piquer-Esteban S, Ruiz-Ruiz S, Arnau V, Diaz W, Moya A. Exploring the universal healthy human gut microbiota around the world. Comput Struct Biotechnol J. 2022;20:421–433. doi:10.1016/j.csbj.2021.12.035. 35035791 PMC8749183

[36] Yao L, Seaton SC, Ndousse-Fetter S, Adhikari AA, Dibenedetto N, Mina AI, Banks AS, Bry L, Devlin ASA. Selective gut bacterial bile salt hydrolase alters host metabolism. eLife. 2018;7:1–32. doi:10.7554/eLife.37001. PMC6078496 30014852

[37] Fiorucci S, Distrutti E. Bile acid-activated receptors, intestinal microbiota, and the treatment of metabolic disorders. Trends Mol Med. 2015;21(11):702–714. doi:10.1016/j.molmed.2015.09.001. 26481828

[38] Cipriani S, Mencarelli A, Chini MG, Distrutti E, Renga B, Bifulco G, Baldelli F, Donini A, Fiorucci S. The bile acid receptor GPBAR-1 (TGR5) modulates integrity of intestinal barrier and immune response to experimental colitis. PLoS One. 2011;6(10):e25637. doi:10.1371/journal.pone.0025637. 22046243 PMC3203117

[39] Stojancevic M, Stankov K. The impact of farnesoid X receptor activation on intestinal permeability in inflammatory bowel disease. Can J Gastroenterol. 2012;26(9):631–637. doi:10.1155/2012/538452. 22993736 PMC3441172

[40] Guinn MLO, Handler ÃDA, Hsieh ÃJJ, Mallicote MU, Feliciano K, Gayer CP. FXR deletion attenuates intestinal barrier dysfunction in murine acute intestinal inflammation. Am J Physiol Gastrointest Liver Physiol. 2024;327:175–187. doi:10.1152/ajpgi.00063.2024. PMC11427094 38860296

[41] Chen T, Xue H, Lin R, Huang Z. MiR-126 impairs the intestinal barrier function via Inhibiting S1PR2 mediated activation of PI3K/AKT signaling pathway. Biochem Biophys Res Commun. 2017;494(3–4):427–432. doi:10.1016/j.bbrc.2017.03.043. 28302479

[42] Zhao S, Gong Z, Du X, Tian C, Wang L, Zhou J, Xu C, Chen Y, Cai W, Wu J. Deoxycholic acid-mediated sphingosine-1-phosphate receptor 2 signaling exacerbates DSS-induced colitis through promoting cathepsin B release. J Immunol Res. 2018. ArtID 2481418. doi:10.1155/2018/2481-418. PMC5966668 29854830

[43] Chiang JYL. Bile acid metabolism and signaling in liver disease and therapy. Liver Res. 2017;1(1):3–9. doi:10.1016/j.livres.2017.05.001.Bile. 29104811 PMC5663306

[44] Nagahashi M, Takabe K, Liu R, Peng K, Wang Y, Hait NC, Wang X, Allegood JC, Aoyagi T, Liang J, et al. Conjugated bile acid activated S1P receptor 2 is a key regulator of sphingosine kinase 2 and hepatic gene expression. Hepatology. 2015;61(4):1216–1226. doi:10.1002/hep.27592.Conjugated. 25363242 PMC4376566

[45] Sharpton SR, Schnabl B, Knight R, Loomba R. Perspective current concepts, opportunities, and challenges of gut microbiome-based personalized medicine in nonalcoholic fatty liver disease. Cell Metab. 2021;33(1):21–32. doi:10.1016/j.cmet.2020.11.010. 33296678 PMC8414992

[46] Sookoian S, Pirola CJ. Liver enzymes, metabolomics and genome-wide association studies: from systems biology to the personalized medicine. World J Gastroenterol. 2015;21(3):711–725. doi:10.3748/wjg.v21.i3.711. 25624707 PMC4299326

[47] Valenzuela-vallejo L, Sanoudou D, Mantzoros CS. Precision medicine in fatty liver disease/non-alcoholic fatty liver disease. J Pers Med. 2023;13:830. doi:10.3390/jpm13050830. 37241000 PMC10224312

[48] Chiang JYL. Bile acids: regulation of synthesis. J Lipid Res. 2009;50(10):1955–1966. doi:10.1194/jlr.R900010-JLR200. 19346330 PMC2739756

[49] Boursier J, Mueller O, Barret M, Machado M, Fizanne L, Araujo-Perez F, Guy CD, Seed PC, Rawls JF, David LA, et al. The severity of nonalcoholic fatty liver disease is associated with gut dysbiosis and shift in the metabolic function of the gut microbiota. Hepatology. 2016;63(3):764–775. doi:10.1002/hep.28356. 26600078 PMC4975935

[50] Wang Y, Leaker B, Qiao G, Sojoodi M, Eissa IR, Epstein ET, Eddy J, Dimowo O, Lauer GM, Chung RT, et al. Precision-cut liver slices as an *ex vivo* model to evaluate antifibrotic therapies for liver fibrosis and cirrhosis. Hepatol Commun. 2024;8:e0558. doi:10.1101/2023.10.30.564772. 39445861 PMC11512631

[51] Leaker BD, Wang Y, Tam J, Anderson RR. Analysis of culture and RNA isolation methods for precision-cut liver slices from cirrhotic rats. Sci Rep. 2024;14(1):1–10. doi:10.1038/s41598-024-66235-2. 38961190 PMC11222550

[52] Lang S, Martin A, Farowski F, Wisplinghoff H, Vehreschild MJGT, Liu J, Krawczyk M, Nowag A, Kretzschmar A, Herweg J, et al. High protein intake is associated with histological disease activity in patients with NAFLD. Hepatol Commun. 2020;4(5):681–695. doi:10.1002/hep4.1509. 32363319 PMC7193126

