# Supplementary material for: Bile salt hydrolase activity as a rational target for MASLD therapy
Source: Gut Microbes. 2026 Jan 2;18(1):2608437. doi: 10.1080/19490976.2025.2608437 (PMC12773562; doi:10.1080/19490976.2025.2608437)
Supplement: Supplementary material [file KGMI_A_2608437_SM0080.docx]

| **Supporting Information**  **Bile salt hydrolase activity as a rational target for MASLD therapy**  Elizabeth V. Jones^a^, Yongtao Wang^b^, Wenchao Wei^c,d^, James C. Reed^b,e^, Snehal N. Chaudhari^a,f^, Darrick K. Li^b,g^, Jerome Boursier^h^, Sonja Lang^c,i^, Münevver Demir^j^, Anna Mae Diehl^k^, Andrew S. Allegretti^l^, Bernd Schnabl^c,d^, Raymond T. Chung,^b,#^ A. Sloan Devlin ^a,#^ |
| --- |

**Supporting Tables**

**Table S1: Patient Characteristics Table, Portal Serum.**

| **patient number** | **age** | **gender** | **race** | **ethnicity** | **antibiotic information** | **etiology of cirrhosis** | **BMI** |
| --- | --- | --- | --- | --- | --- | --- | --- |
| PS-01 | 72.10 | male | Caucasian | not Hispanic | none | MASH | 30.22 |
| PS-02 | 59.80 | male | Caucasian | not Hispanic | Cipro | Alcohol Liver Disease | 34.04 |
| PS-03 | 74.21 | female | Caucasian | not Hispanic | none | Mixed | 23.12 |
| PS-04 | 70.34 | male | Caucasian | not Hispanic | none | Alcohol Liver Disease | 26.12 |
| PS-05 | 56.80 | male | Caucasian | not Hispanic | Ceftriaxone | Mixed | 28.49 |
| PS-06 | 57.53 | male | Caucasian | not Hispanic | Cipro | Mixed | 25.69 |
| PS-07 | 51.37 | female | Caucasian | not Hispanic | none | Alcohol Liver Disease | 19.94 |
| PS-08 | 58.74 | male | Caucasian | not Hispanic | none | MASH | 29.49 |
| PS-09 | 52.73 | male | Caucasian | not Hispanic | none | Alcohol Liver Disease | 28.12 |
| PS-10 | 67.62 | male | Caucasian | not Hispanic | Cipro | Alcohol Liver Disease | 18.65 |
| PS-11 | 41.04 | female | Caucasian | not Hispanic | none | Mixed | 33.06 |
| PS-12 | 57.38 | female | Caucasian | not Hispanic | none | Mixed | 22.43 |
| PS-13 | 61.23 | male | Caucasian | not Hispanic | none | Other | 30.85 |
| PS-14 | 71.97 | male | Caucasian | not Hispanic | none | Alcohol Liver Disease | 28.41 |
| PS-15 | 50.39 | female | Caucasian | not Hispanic | none | MASH | 36.46 |
| PS-16 | 62.44 | female | Caucasian | not Hispanic | none | Other | 25.50 |

**Table S2: Literature reports of portal bile acids in humans without liver disease.**

Reported portal serum bile acid concentrations in patients with uncomplicated gallstone disease or elective cholecystectomy with normal liver function. Range from these studies are 6 – 22 µM total bile acids quantified,^37–40^ compared to average total bile acid concentration of 116 µM in our cohort of advanced liver disease patients.

| **Source** | **N** | **Patient Status** | **total bile acids (average)** | **Specific Bile Acids Quantified (average)** |
| --- | --- | --- | --- | --- |
| Schersten  J. Gastroenterology  1977 | 26 | uncomplicated gallstone disease (controls) | 12.9 ± 1.5 µmol/L |  |
| Ewerth  Hepatology  1985 | 9 | Elective cholecystectomy, gallstone free; normal liver function | 17.86 µmol/L | CAs: 7.02 ± 3.03 µmol/L  CDCAs: 7.23 ± 2.80 µmol/L  DCAs: 3.61 ± 1.05 µmol/L |
| Einarsson Gastroenterology  1977 | 10 | Elective cholecystectomy from uncomplicated gallstone disease; normal liver function | 22.16 µmol/L | CAs: 6.14 ± 1.20 µmol/L  CDCAs: 8.40 ± 1.84 µmol/L  DCAs: 7.62 ± 2.76 µmol/L |
| Hesselfeldt  Scan. J. Gastroenterology  1984 | 8 | Elective cholecystectomy from uncomplicated gallstone disease; normal liver function | 6.67 µmol/L | GCA: 2.09 µmol/L  GCDCA: 1.24 µmol/L  GDCA: 0.84 µmol/L  TCA: 1.16 µmol/L  TCDCA: 0.85 µmol/L  TDCA: 0.49 µmol/L |
| Note: CAs/CDCAs/DCAs refers to the sum of unconjugated and conjugated versions of the bile acid, as the treatment involved conditions that hydrolyzed the amide bond in conjugated bile acids. | | | | |

**Table S3: Patient Characteristics Table, Fecal Samples.**

| Patient number | Age | Sex | Steatosis grade | Lobular inflammation | Hepatocyte ballooning | MAS score | Fibrosis Score |
| --- | --- | --- | --- | --- | --- | --- | --- |
| 1 | 65.23 | male | 0 | 0 | 0 | 0 | 4 |
| 2 | 69.00 | male | 2 | 1 | 1 | 4 | 2 |
| 3 | 73.04 | male | 2 | 2 | 2 | 6 | 3 |
| 4 | 49.95 | male | 2 | 1 | 1 | 4 | 1 |
| 5 | 40.91 | male | 2 | 0 | 0 | 2 | 0 |
| 6 | 62.96 | female | 1 | 1 | 0 | 2 | 1 |
| 7 | 54.87 | female | 3 | 1 | 2 | 6 | 1 |
| 8 | 53.16 | female | 1 | 1 | 1 | 3 | 0 |
| 9 | 21.57 | male | 2 | 1 | 0 | 3 | 0 |
| 10 | 53.68 | female | 1 | 1 | 0 | 2 | 0 |
| 11 | 41.53 | female | 1 | 1 | 0 | 2 | 0 |
| 12 | 63.64 | male | 3 | 1 | 0 | 4 | 0 |
| 13 | 62.46 | female | 1 | 1 | 0 | 2 | 0 |
| 14 | 71.67 | female | 0 | 1 | 0 | 1 | 0 |
| 15 | 76.44 | female | 2 | 1 | 1 | 4 | 1 |
| 16 | 53.20 | male | 1 | 1 | 1 | 3 | 1 |
| 17 | 58.15 | female | 2 | 1 | 1 | 4 | 1 |
| 18 | 57.24 | male | 1 | 1 | 0 | 2 | 1 |
| 19 | 65.01 | male | 1 | 1 | 1 | 3 | 1 |
| 20 | 36.89 | female | 0 | 0 | 0 | 0 | 1 |
| 21 | 70.79 | male | 3 | 1 | 2 | 6 | 2 |
| 22 | 45.64 | male | 3 | 1 | 1 | 5 | 2 |
| 23 | 59.99 | male | 1 | 1 | 1 | 3 | 2 |
| 24 | 62.92 | male | 3 | 1 | 2 | 6 | 2 |
| 25 | 38.15 | male | 3 | 1 | 1 | 5 | 3 |
| 26 | 61.52 | male | 3 | 2 | 2 | 7 | 3 |
| 27 | 67.30 | female | 1 | 1 | 1 | 3 | 3 |
| 28 | 63.86 | male | 1 | 1 | 0 | 2 | 3 |
| 29 | 51.59 | female | 2 | 2 | 2 | 6 | 4 |
| 30 | 61.89 | male | 1 | 1 | 1 | 3 | 4 |
| 31 | 59.26 | female | 1 | 1 | 2 | 4 | 4 |
| 32 | 24.86 | female | 1 | 1 | 1 | 3 | 4 |
| 33 | 78.85 | male | 1 | 1 | 1 | 3 | 1 |
| 34 | 45.59 | male | 1 | 1 | 0 | 2 | 1 |
| 35 | 64.32 | male | 1 | 1 | 0 | 2 | 1 |
| 36 | 63.09 | female | 3 | 1 | 1 | 5 | 1 |
| 37 | 41.46 | male | 0 | 1 | 0 | 1 | 0 |
| 38 | 71.19 | female | 0 | 1 | 0 | 1 | 0 |
| 39 | 67.14 | male | 2 | 2 | 0 | 4 | 0 |
| 40 | 48.76 | male | 2 | 1 | 0 | 3 | 0 |
| Patient number | Age | Sex | Steatosis grade | Lobular inflammation | Hepatocyte ballooning | MAS score | Fibrosis Score |
| 41 | 51.12 | male | 0 | 1 | 0 | 1 | 0 |
| 42 | 55.84 | female | 3 | 2 | 1 | 6 | 1 |
| 43 | 52.07 | male | 1 | 1 | 0 | 2 | 0 |
| 44 | 47.37 | male | 1 | 0 | 0 | 1 | 1 |
| 45 | 49.63 | male | 0 | 0 | 0 | 0 | 0 |
| 46 | 69.68 | female | 1 | 1 | 0 | 2 | 0 |
| 47 | 46.61 | male | 1 | 1 | 0 | 2 | 1 |
| 48 | 61.59 | female | 0 | 0 | 0 | 0 | 0 |
| 49 | 50.03 | male | 0 | 0 | 0 | 0 | 0 |
| 50 | 49.92 | male | 2 | 1 | 1 | 4 | 1 |
| 51 | 58.88 | female | 1 | 1 | 0 | 2 | 0 |
| 52 | 56.66 | female | 2 | 1 | 2 | 5 | 4 |
| 53 | 64.84 | female | 3 | 2 | 2 | 7 | 2 |
| 54 | 68.42 | female | 1 | 1 | 2 | 4 | 2 |
| 55 | 58.07 | male | 2 | 2 | 1 | 5 | 2 |
| 56 | 55.54 | male | 3 | 2 | 2 | 7 | 2 |
| 57 | 45.56 | male | 2 | 2 | 2 | 6 | 2 |
| 58 | 59.56 | female | 1 | 1 | 2 | 4 | 2 |
| 59 | 58.61 | male | 1 | 1 | 1 | 3 | 2 |
| 60 | 62.12 | male | 3 | 2 | 2 | 7 | 2 |
| 61 | 62.83 | female | 1 | 3 | 2 | 6 | 3 |
| 62 | 51.21 | female | 3 | 2 | 2 | 7 | 3 |
| 63 | 70.23 | female | 1 | 1 | 1 | 3 | 2 |
| 64 | 76.24 | female | 2 | 2 | 2 | 6 | 4 |
| 65 | 78.84 | female | 1 | 2 | 2 | 5 | 3 |
| 66 | 63.63 | female | 3 | 2 | 2 | 7 | 4 |
| 67 | 55.66 | female | 2 | 1 | 1 | 4 | 4 |
| 68 | 67.84 | male | 1 | 1 | 1 | 3 | 4 |
| 69 | 61.09 | male | 2 | 1 | 1 | 4 | 4 |
| 70 | 58.12 | male | nd | nd | nd | 0 | nd |
| 71 | 67.68 | male | nd | nd | nd | 0 | nd |
| 72 | 60.22 | female | 3 | 2 | 2 | 7 | 4 |
| 73 | 29.84 | male | 1 | 2 | 0 | 3 | 4 |
| 74 | 69.00 | female | nd | nd | nd | 0 | nd |
| 75 | 56.32 | male | nd | nd | nd | 0 | nd |
| 76 | 64.71 | female | nd | nd | nd | 0 | nd |
| 77 | 79.61 | female | nd | nd | nd | 0 | nd |
| 78 | 53.91 | male | 3 | 3 | 2 | 8 | 4 |
| 79 | 66.15 | female | nd | nd | nd | 0 | nd |
| 80 | 58.95 | female | 2 | 2 | 2 | 6 | 4 |
| 81 | 64.03 | male | nd | nd | nd | 0 | nd |
| Patient number | Age | Sex | Steatosis grade | Lobular inflammation | Hepatocyte ballooning | MAS score | Fibrosis Score |
| 82 | 64.74 | female | 3 | 2 | 1 | 6 | 0 |
| 83 | 32.41 | female | 2 | 1 | 0 | 3 | 0 |
| 84 | 52.25 | female | 2 | 1 | 0 | 3 | 0 |
| 85 | 63.55 | male | 1 | 0 | 1 | 2 | 0 |
| 86 | 29.23 | male | 2 | 0 | 0 | 2 | 0 |
| 87 | 51.89 | female | 2 | 1 | 0 | 3 | 0 |
| 88 | 35.58 | female | 2 | 1 | 1 | 4 | 0 |
| 89 | 44.28 | male | 1 | 1 | 1 | 3 | 0 |
| 90 | 52.96 | female | 2 | 1 | 1 | 4 | 0 |
| 91 | 62.37 | male | 2 | 0 | 0 | 2 | 0 |
| 92 | 53.30 | female | 1 | 1 | 1 | 3 | 0 |
| 93 | 67.17 | female | 3 | 0 | 0 | 3 | 0 |
| 94 | 28.76 | female | 3 | 0 | 0 | 3 | 0 |
| 95 | 37.71 | male | 1 | 1 | 0 | 2 | 0 |
| 96 | 39.37 | male | 1 | 1 | 1 | 3 | 1 |
| 97 | 42.20 | female | 1 | 1 | 1 | 3 | 1 |
| 98 | 31.94 | male | 3 | 1 | 1 | 5 | 1 |
| 99 | 52.26 | female | 1 | 1 | 1 | 3 | 1 |
| 100 | 28.96 | male | 1 | 1 | 0 | 2 | 1 |
| 101 | 59.31 | female | 2 | 1 | 1 | 4 | 1 |
| 102 | 60.76 | male | 3 | 1 | 2 | 6 | 1 |
| 103 | 30.05 | female | 3 | 1 | 1 | 5 | 1 |
| 104 | 28.24 | male | 3 | 0 | 0 | 3 | 1 |
| 105 | 55.64 | female | 2 | 2 | 1 | 5 | 1 |
| 106 | 41.66 | female | 2 | 2 | 1 | 5 | 1 |
| 107 | 20.18 | female | 3 | 1 | 1 | 5 | 1 |
| 108 | 57.86 | female | 3 | 1 | 1 | 5 | 1 |
| 109 | 69.59 | female | 1 | 2 | 2 | 5 | 1 |
| 110 | 58.93 | female | 2 | 1 | 2 | 5 | 1 |
| 111 | 32.51 | male | 1 | 2 | 1 | 4 | 1 |

**Table S4: Aggregate data of BSH activity and 16S results from isolates of patients 1, 2, and 3, may be found in a separate excel file “Table S4.”**

**Table S5: RT-qPCR primers**

| **Sequences** | **Targets** | Source | **Species** |
| --- | --- | --- | --- |
| ATGACTCTACCCACGGCAAG | *GAPDH*-F | **Invitrogen** | Human |
| CTGGAAGATGGTGATGGGTT | *GAPDH*-R | **Invitrogen** | Human |
| CAATTCCTGGCGATACCTCAG | *TGFB1*-F | **Invitrogen** | Human |
| GCACAACTCCGGTGACATCAA | ***TGFB1*-R** | **Invitrogen** | Human |
| AAAAGACAGCTACGTGGGTGA | *ACTA2*-F | **Invitrogen** | Human |
| GCCATGTTCTATCGGGTACTTC | *ACTA2*-R | **Invitrogen** | Human |
| CAGCCGCTTCACCTACAGC | *COL1A1*-F | **Invitrogen** | Human |
| TCAATCACTGTCTTGCCCCA | *COL1A1*-R | **Invitrogen** | Human |
| ACTTCCACAGGTCCCACAAC | *TIMP1*-F | **Invitrogen** | Human |
| GCTAAGCTCAGGCTGTTCCA | *TIMP1*-R | **Invitrogen** | Human |
| TTCGGTCCAGTTGCCTTCTC | *IL6*-F | **Invitrogen** | Human |
| TCTTCTCCTGGGGGTACTGG | *IL6*-R | **Invitrogen** | Human |

**Supporting Figures**

**Figure S1.** **Bile acid concentrations in portal veins of patients undergoing TIPS procedure**. Bile acids were quantified using UPLC-MS. All bile acids with measurable concentrations above the limit of detection are shown. Data are shown as mean ± SEM.

**
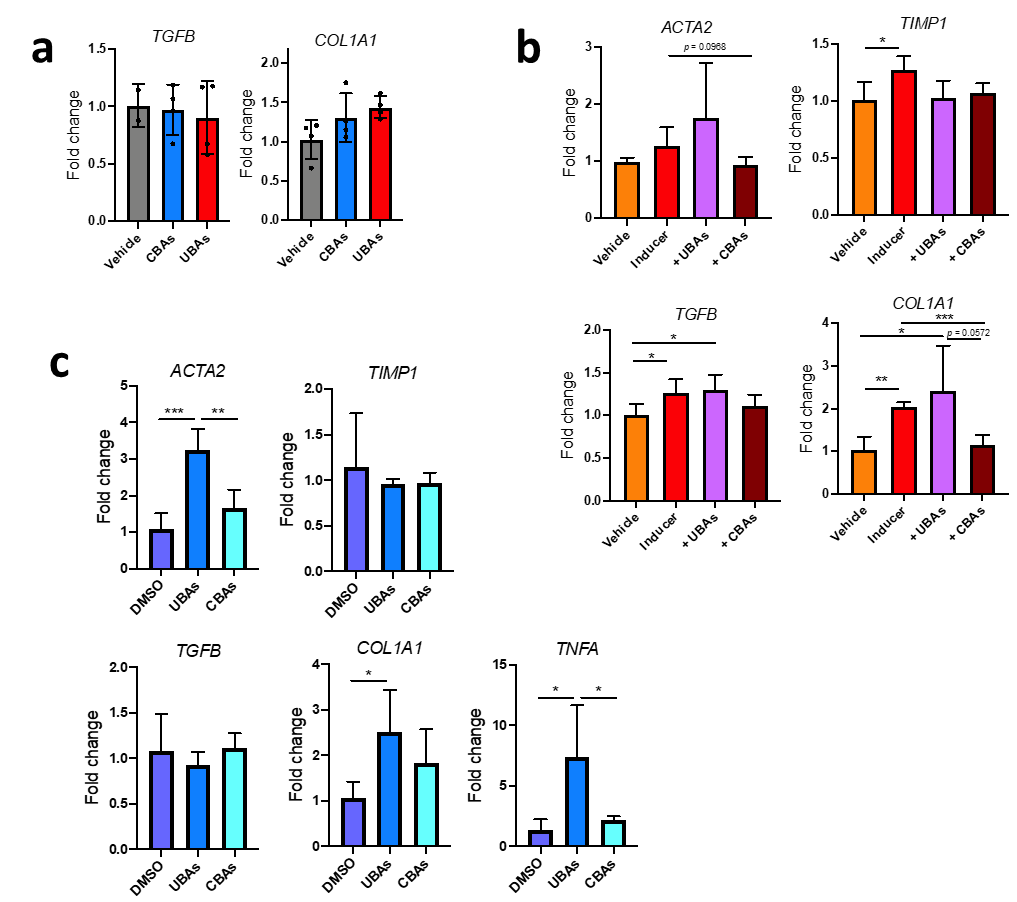
**

**Figure S2:** Unconjugated bile acid pools heighten pro-fibrogenic responses in liver cells. A) PCLS from human MASLD were treated with 50 μM CBAs or 50 μM UBAs for 48 hrs, followed by qPCR. B) pHSC cells were treated with 10 ng/mL TGFβ (inducer) for 24 hrs, then treated with 250 μM tauro-CBAs and/or 250 μM UBAs for 48 hrs. C) LX2 cells were treated with a pool of CBAs, a pool of UBAs at 250 μM, or DMSO for 48 hrs, followed by qPCR. Data are shown as mean ± SEM. The CBA pool consisted of a 3:1 mixture of glyco bile acids (GCA, GCDCA, GUDCA, GDCA, GLCA in equal proportion) to tauro bile acids (TCA, TCDCA, TUDCA, TDCA, TLCA in equal proportion). UBA pool consisted of CA, CDCA, UDCA, DCA, LCA in equal proportion. (One-way ANOVA, n=4 biological replicates per group for all experiments. The experiments were repeated 3 times with similar results.)


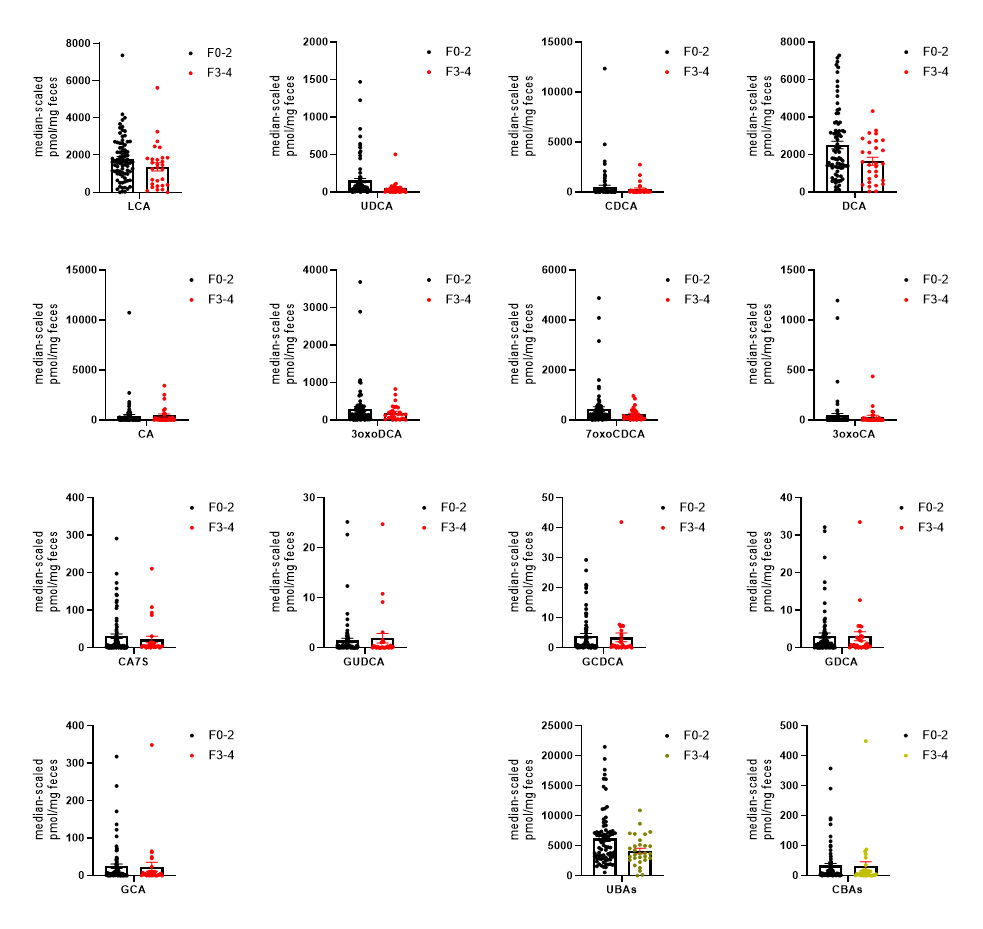


**Figure S3.** **Bile acid concentrations in feces of MASLD patients, grouped by patient F stage**. Bile acids were quantified using UPLC-MS. All bile acids with measurable concentrations above the limit of detection are shown. Data are shown as mean ± SEM.


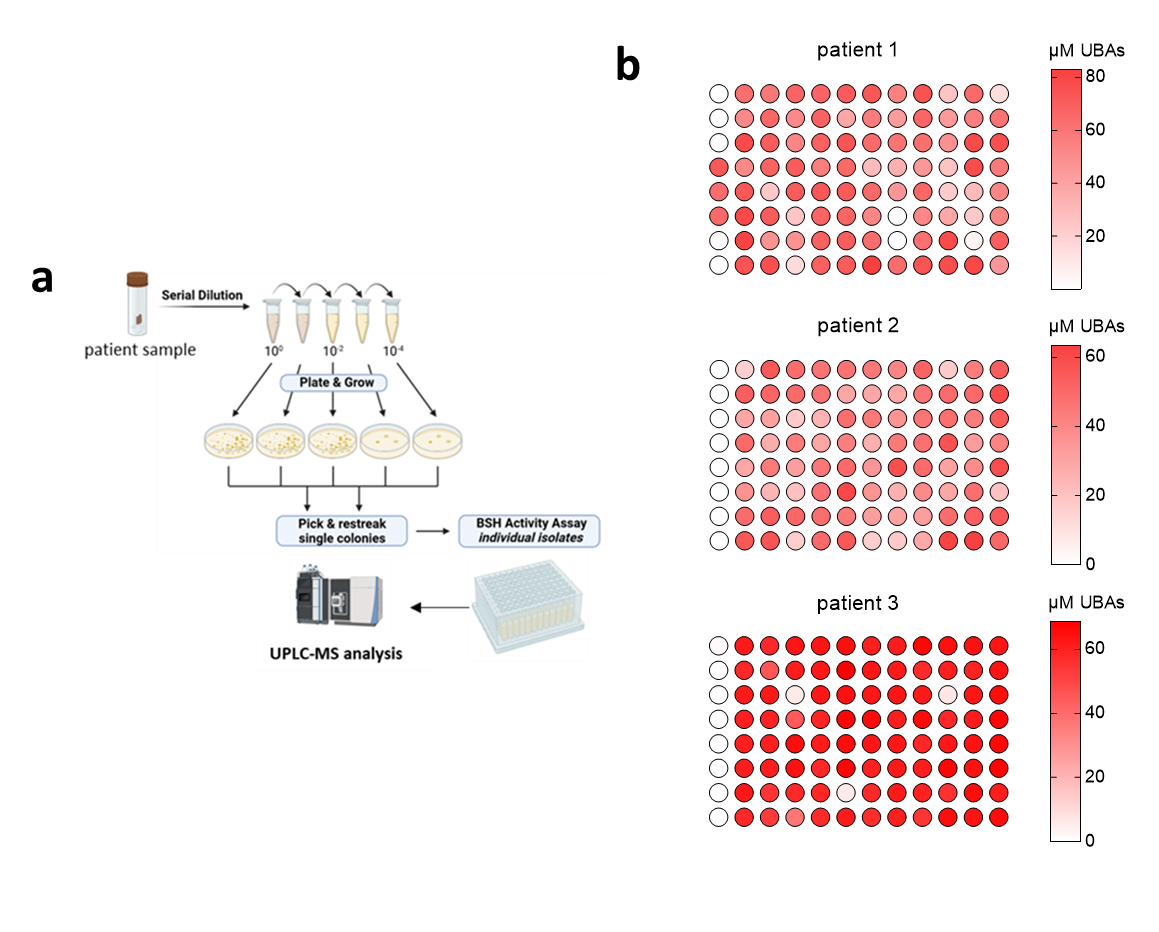


**Figure S4. BSH activity screening of MASLD patient fecal isolates** A) Schematic of single colony isolate picking and BSH activity screening assay workflow. A frozen fecal patient sample was anaerobically resuspended in anaerobicized PBS to 5 mg of feces/ml of PBS. Fecal slurry was serial diluted and plated on BHI+ agar and incubated 37 °C for 2 days. Individual colonies were picked, restreaked onto new BHI+ plates, and incubated 37 °C for 2 days. Pure isolates were moved into 96-deep well plate with 600 µL BHI+ media and grown overnight. Saturated cultures were diluted 1:10 into fresh BHI+ media containing 50 µM each of GLCA and GCA and incubated 37 °C for 24 h. Resultant supernatant was extracted for UPLC-MS analysis. B) Concentrations of LCA+CA quantified via UPLC-MS per patient well/plate. BSH assays were performed once in singlicate (one culture per isolate) given the high volume of isolates.


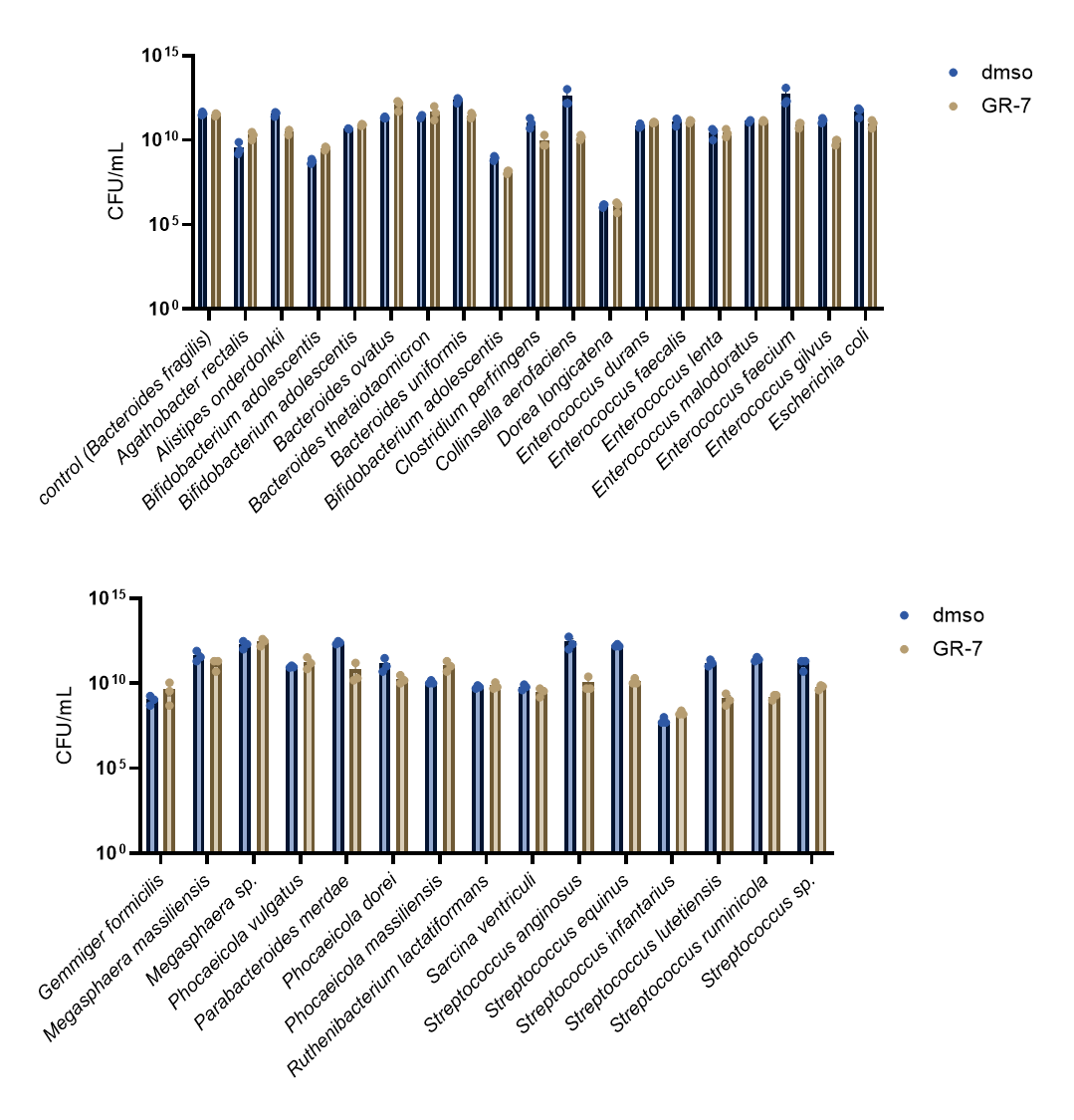


**Figure S5. GR-7 has no adverse effects on bacterial viability.** No substantial growth inhibition was observed when MASLD isolates were treated with the BSH inhibitor GR-7. Plating was performed in biological triplicate for each condition. CFU, colony-forming units. Three biological replicates per group, experiments performed once due to workflow throughput constraints. Data are shown as mean ± SEM.
